# Supplementary figures and images for: PEREGRINE: A genome-wide prediction of enhancer to gene relationships supported by experimental evidence
Source: PLoS One. 2020 Dec 15;15(12):e0243791. doi: 10.1371/journal.pone.0243791 (PMC7737992; doi:10.1371/journal.pone.0243791)

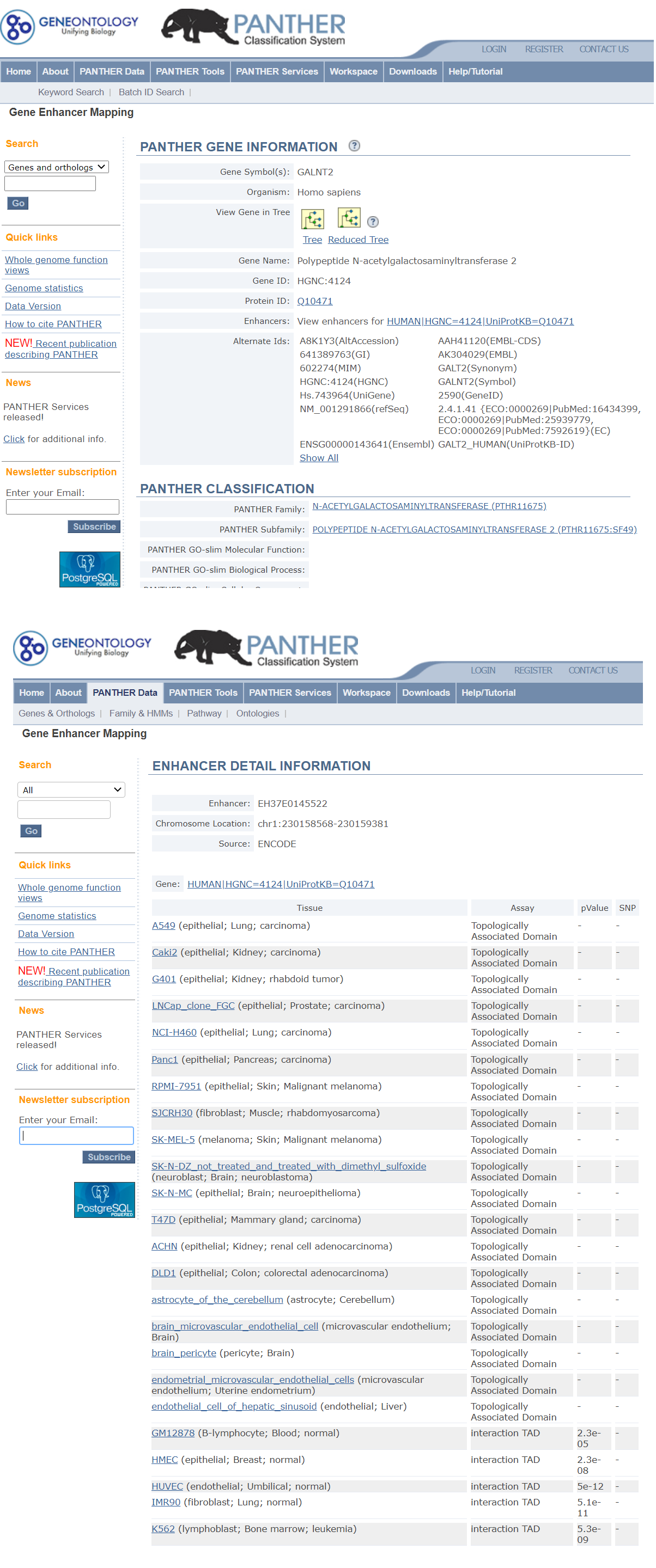

Supplement: S1 Fig — a. Gene detail page. b. Enhancer detail page. (TIF) [file pone.0243791.s001.tif]
