# Supplementary material for: PEREGRINE: A genome-wide prediction of enhancer to gene relationships supported by experimental evidence
Source: PLoS One. 2020 Dec 15;15(12):e0243791. doi: 10.1371/journal.pone.0243791 (PMC7737992; doi:10.1371/journal.pone.0243791)
Supplement: S1 File — Download URLs for enhancer sets from original sources. (PDF) [file pone.0243791.s002.pdf]

URLs for data downloads

ENCODE's catalog of candidate Cis-Regulatory Elements (https://www.encodeproject.org/files/ENCFF788SJC/@@download/ENCFF788SJC.bed.gz)

VISTA (https://enhancer.lbl.gov/cgi-bin/imagedb3.pl?show=1;page=1;form=ext_search;search.status=Positives;search.org=Human;page_size=100;search.result=yes;action=search;search.gene=;search.sequence=1)

Ensembl (http://grch37.ensembl.org/biomart/martview/440f9aee4e5507d9e90a756bcf8a3f4c)

FANTOM (http://slidebase.binf.ku.dk/human_enhancers/).
