# Supplementary material for: PEREGRINE: A genome-wide prediction of enhancer to gene relationships supported by experimental evidence
Source: PLoS One. 2020 Dec 15;15(12):e0243791. doi: 10.1371/journal.pone.0243791 (PMC7737992; doi:10.1371/journal.pone.0243791)
Supplement: Attachment — Submitted filename: Response to Reviewers.docx [file pone.0243791.s003.docx]

Oct. 25, 2020

Dear Dr. Ludmila Prokunina-Olsson,

We thank you and the reviewers very much for the thorough reading of our manuscript and for the helpful critiques. Enclosed please find our revised manuscript. Since the manuscript was originally submitted to PLOS Computational Biology, it was formatted according to its guidelines. In this revision, the following format updates were made to meet the guidelines of PLOS One.

1. The “Author summary” section was deleted.
2. The “Materials and methods” section was moved up to right after the “Introduction” section.
3. The “Results” and “Discussion” sections in the original manuscript were combined into the “Results and discussion” section in the revised manuscript.
4. A “Conclusion” section was added.

Due to the reformat, as well as other updates suggested by the reviewers, the figure, table and line numbers are different in the revised manuscript compared to the original one. In our response below, I will make sure to specify the version of manuscript used, and if needed, use numbers from both versions.

Below are the specific responses to each of the reviewers’ comments. Our responses are in blue. The modified texts are highlighted in red in the “Revised Manuscript with Track Changes”.

*Reviewer #1:*

*Minor Revisions:*

*• The authors define in the introduction what typically denotes an enhancer region, but the criteria used in the individual datasets to classify a non-coding element as an enhancer. i.e., H3K27ac, DHS, H3K4me1, TF binding, etc, should be acknowledged in the methods as there might be variation amongst the datasets.*

We thank the reviewer for pointing it out. We clarify this by modify the description in the Materials and methods section (line 159-163 in the revised manuscript) as below:

“These enhancer coordinates were the result of experiments performed across various tissues by their respective sources, which differed somewhat according to each enhancer source. We did not filter out any enhancers from these sources, instead opting to utilize their differing enhancer calling pipelines simultaneously to maximize the chance of capturing the highest number of enhancers.”

*• It is unclear how the expected number of links was determined for the GO analysis?*

We appreciate reviewer’s comment here. We clarified the meaning of “expected” under the subsection of “Statistical Analysis” in “Materials and Methods” section (line 248-249 in the revised manuscript) by rephrasing the sentence as below:

“We performed enrichment analysis using Gene Ontology Biological Processes if there were any gene pathways enriched by having more or less than the expected number of linked enhancers per gene under the null hypothesis of the Mann Whitney U test that the two samples come from the same distribution via the PANTHER web interface.”

*• Line 209: Table 4 caption indicates that the most target genes an enhancer has is 17, but the table indicates 34.*

We apologize for the mistakes here. We corrected the number to 34 in line 306 in the revised manuscript.

*• Lines 210-211: Table 4 caption states that over 77% of enhancers are linked to only 1 gene, but the table and text suggest 56% of enhancers have 1 gene, 78% of enhancers have either 1 or 2 genes linked to it.*

We corrected the number to 56% in line 306 in the revised manuscript.

*• Line 211-212: Table 4 caption states >97% of enhancers have 4 or less target genes, this is not what is represented in the text or table. 93% of enhancers have 4 or less target genes, 97% of enhancers have 6 or less target genes.*

We corrected the text to “97% of enhancers here were found to have six or less target genes” in line 308 in the revised manuscript.

*• Figure 5 is hard to distinguish the genes from the enhancers. Additionally, in the UCSC Genome Browser, the browser image can be viewed by right clicking the browser and saved rather than a browser screen shot.*

We thank the reviewer for the suggestion. The figures (Figure 3 in the revised manuscript) were regenerated with the suggested method.

*• Lines 263-266: Figure 5 caption b) does not match the figure, it should be chromosome 14*

We corrected the caption for Figure 5b to “chromosome 14” in line 348 of the revised manuscript.

*• It is not clear to this reviewer what is gained by Figure 6 as it is the same data in figure 4 but in bins of 5.*

We take the reviewer’s advice and removed the figure from the manuscript.

*• Please ensure that the appropriate nomenclature is used for gene and protein names.*

We appreciate reviewer’s comment. Under the subsection of “Utilizing the PEREGRINE data in PANTHER”, examples of genes and their relationships to the enhancers were described. All the gene names on page 21-23 of the revised manuscript are italicized according to the gene nomenclature guideline.

*• Figure 9, the label position for the non-PEREGRINE enhancer datasets is poorly placed inside the PEREGRINE enhancer circle. In this reviewer's opinion, it made interpretation confusing.*

The figure (Figure 6 in the revised manuscript) labels were updated to make it more clear about the non-PEREGRINE sections.

*Reviewer #2:*

*While for the most part the manuscript is technically sound, there are instances throughout where table and figure legends do not agree with the data in the table or figure (e.g. Table 4).*

We thank reviewer’s comment. We updated the legends for Table 4 and Figure 3 in the revised manuscript (Figure 5 in the original version) (see our response to reviewer 1 above). We also reviewed all the other figure and table legends to make sure that the descriptions are accurate.

*Multiple figures and tables which largely show the same data seems unnecessary and could be condensed (fig 2, 3 and table 4).*

This is a good suggestion. We combined Figures 2, 3, 4 in the original manuscript to one figure, Figure 2 in the revised one.

*Screenshots of UCSC genome browser views are not the most appropriate figure when the same tool allows for the output of figures as PDF without any of the online user interface as part of the final figure.*

The figure (Figure 5 in the original manuscript, and Figure 3 in the revised one) was regenerated according to the suggested method.

We thank you and the reviewers again for the helpful suggestions and comments.

Sincerely,

Huaiyu Mi, Ph.D.

Department of Preventive Medicine

Keck School of Medicine

University of Southern California
